# Empowering a Limpopo rural community to Integrate Ubuntu in caring for PLWHIV/AIDS or TB

**DOI:** 10.4102/curationis.v47i2.2628

**Published:** 2024-12-03

**Authors:** Thifhelimbilu I. Ramavhoya, Mamoeng N. Kgatla, Molatelo M. Rasweswe, Fhumulani M. Mulaudzi

**Affiliations:** 1 Department of Nursing Science, Faculty of Health Sciences, University of Limpopo, Polokwane, South Africa; 2 Department of Nursing Science, Faculty of Health Sciences, University of Pretoria, Pretoria, South Africa

**Keywords:** empowerment, UBUNTU, caring, integrating, community, HIV/AIDS, TB

## Abstract

**Background:**

Globally, few studies were conducted mostly in Africa on Ubuntu and human immunodeficiency virus/acquired immunodeficiency syndrome (HIV/AIDS). These studies did not incorporate empowering communities on integrating Ubuntu HIV/AIDS or tuberculosis (TB). As such, this study used empowerment as a tool to raise awareness in the community about how they can integrate Ubuntu when caring for people living with HIV (PLWHIV)/AIDS or TB.

**Objectives:**

This study aimed to empower community members to use Ubuntu philosophy when caring for PLWHIV/AIDS or TB in the rural community of Limpopo province.

**Method:**

An integrative qualitative research design was used in which data were collected through a workshop with participants. The population was unemployed matriculants, traditional health professionals and community healthcare workers who were purposefully sampled and divided into eight groups. Data were collected through a focus group discussion and analysed using content analysis. Ethical considerations and measures to ensure trustworthiness were followed.

**Results:**

Integrating Ubuntu philosophy can reduce stigma, discrimination and lack of disclosure. This might be achieved through humanity, attitude changing and formation of a support group.

**Conclusion:**

The principles of Ubuntu (caring, sharing, support, humility, attitude change) came up strongly in preventing stigma and discrimination in the care of PLWHIV/AIDS or TB.

**Contribution:**

Community empowerment and integration of Ubuntu philosophical values might have a positive impact on improving the social status of PLWHIV or TB, their families and the community at large.

## Introduction

Empowerment is one of the strategies used to capacitate individuals and the community to be involved and take ownership of their own health. Empowerment is a process and is seen as a fundamental tool to community development; as such, it promotes both subjective well-being and objective changes to its community, hence bringing the desired changes to their health (Leong et al. 2019). Empowerment improves local power and well-being of the targeted community, leading to change in attitude and behaviour (Hanke & Lowitzsch 2020). As such, in this instance, it was the result of interpersonal, mutual and collective social action where the community was taught how to integrate the Ubuntu philosophy in caring, preventing and managing the spread of human immunodeficiency virus (HIV) and acquired immunodeficiency syndrome (AIDS) or tuberculosis (TB). Since the beginning of the pandemic, HIV in 1959 in Khinshasa, mid-to-late 1970s in the United Kingdom and 1981 in African countries, including South Africa, people living with the condition have been stigmatised and discriminated in one way or another (Rasweswe et al. 2024; Sharp & Hahn 2011). If stigmatisation and discrimination are not addressed, it could lead to depression and anxiety in people living with human immunodeficiency virus (PLWHIV) (Dejman et al. 2017; Fauk et al. 2021). Although the country is being made aware of HIV/AIDS annually through World AIDS Day, it was observed that those affected are still stigmatised and Ubuntu philosophy is no longer practised, hence this workshop was conducted for the participants to instil the principles of Ubuntu when caring for PLWHIV/AIDS or TB.

Ubuntu is an indigenous African philosophy and way of life that has been used for many centuries to shape, guide and maintain positive human interactions, relationships and well-being among African indigenous people and communities (Ramose 2002). The word ‘Ubuntu’ was obtained from the word ‘muthu’, which means a human being in indigenous languages (Venda), the southern African Nguni group of languages (isiZulu/isiXhosa/isiSwati/isiNdbele). ‘Umuntu’ is ‘motho’ in the SeSotho language, another indigenous South African language (Mulaudzi et al. 2022). There is no Ubuntu before there is a human being, ‘umuntu’, or human beings, ‘abantu’ (Mbeje 2010). Mbeje (2010) points out that in Africa, a human being lives among other human beings to form a family, a community or a society. Nxumalo and Mncube (2018) used Collective Fingers Theory to explore the value of incorporating Ubuntu philosophy into everyday lives, activities and circumstances of people, which includes conditions such as HIV/AIDS and TB. Therefore, studies on Ubuntu and HIV/AIDS were conducted worldwide, mostly in African countries. For example, Tarkang, Pencille and Komesuor (2018) conducted a systemic review in Africa on the applicability of the *Ubuntu* concept to promote safe sexual practices and positive attitudes towards PLWHIV. Another study was conducted by Racheal (2016) to establish how the Ubuntu principle is reflective in Shona-speaking people as they promote the well-being of HIV/AIDS orphaned learners living in Masvingo City in Zimbabwe. The findings of their study revealed that extended families applied Ubuntu by helping orphans with physical needs so that their health could be improved. In South Africa, Mulaudzi (2012) explored the experiences of Batswana families with respect to hospice care for AIDS patients, where family members had mixed feelings in relation to care of their loved ones in the hospice, as they believed in the principles of Ubuntu as Africans where one must take care of one another through thick and thin. Another study was conducted in Limpopo province (Rasweswe et al. 2024), where Ubuntu was considered as a critical component that could be used in the fight against HIV/AIDS and TB stigma. In their study, the authors discovered that the Ubuntu principles and their values, which include respect, treating people with dignity, love, compassion, caring, if practised can reduce the stigma associated with people living with the virus and their family members. The same was indicated in the article written by Netshisaulu and Makhema (2021) in Limpopo province, where the authors emphasised the integration of Ubuntu philosophical values into disease prevention and management, which can promote case identification and adherence to treatment. Mbokazi et al. (2023) concurred that Ubuntu is a mediator in coping with multimorbidity treatment burden in a disadvantaged rural and urban setting of Limpopo province, South Africa. However, these studies did not include the empowerment of the community to integrate Ubuntu into the care of people living with HIV (PLWHIV)/AIDS or TB. Therefore, this training was conducted to empower the community in order to integrate Ubuntu when interacting, caring and managing PLWHIV or TB. These will improve the quality of life of PLWHIV/AIDS or TB.

### Setting

The workshop was held at Shotong community hall with the community, Ga Kgapane, Greater Letaba subdistrict, Mopani. Mopani is one of the five districts located in Limpopo province. It has a population of 1 150 722, including children over 5 years of age. The district has 41% of PLWHIV, along with others who have co-infections with TB. Like any other province, new infections are being detected daily because of its borders with Zimbabwe and Mozambique. There is an estimate of 80 834 matriculants who are unemployed with an increase in the emerging number of traditional healthcare practitioners. The district consists of community healthcare workers (CHCWs) with an estimated of 20 attached to each primary healthcare (PHC) facility. Community healthcare workers visit patients at home and refer them to healthcare facilities on a daily basis.

## Research methods and design

### Research design

An integrative qualitative research design was used in which participants were divided into eight groups. This was done to facilitate participation among the groups so that they can demonstrate how they can integrate Ubuntu Philosophical principles and its values when interacting with PLWHIV/AIDS or TB.

### Population and sampling

For this workshop, the population included unemployed matriculants, traditional health practitioners and CHCWs. A total of 152 participants were purposively selected because the target for this training was aimed at CHCWs, traditional health practitioners and unemployed matriculants. The reason for selection was to empower them with knowledge because traditional health practitioners and CHCWs are the first people to be in contact with the community. For unemployed matriculants, the purpose of selecting them was to make them employable in PHC services so that they assist nurses by practising and instilling Ubuntu in the community so that they accept and care for PLWHIV/AIDS or TB.

### Data collection

An interactive workshop was conducted for 5 days in February 2023 with the community and three facilitators who are experts on HIV/AIDS, TB and Ubuntu. Among the facilitators, two worked in PHC facilities for more than 15 years, practising and providing HIV/AIDS and TB services. Of the two who worked in PHC facilities, one was a trainee trainer for both conditions. Lastly, the third one was an expert on Ubuntu. In attendance, there were 152 participants, who were divided into eight focus groups to facilitate the group and ensure that all participated in the activities during the workshop. During the first 3 days of the workshop, facilitators provided information on Ubuntu, HIV/AIDS and TB. Within these days, various activities were assigned to groups for them to present back to the large audience with clarification from facilitators and other participants. This article presented a portion of the work that was allocated to groups on the fourth day. The following are topics that were allocated to the groups to present. From group 1–4, topics on HIV were assigned as follows: (1) implementing Ubuntu when caring for PLWHIV, (2) use of Ubuntu to prevent stigma and discrimination in PLWHIV, (3) integrating Ubuntu in the prevention of HIV and (4) integrating Ubuntu in the management of HIV. The same topics were assigned to groups 5–8, but with a focus on TB. The groups were given 45 min to deliberate on their topic. A marker and a flip chart were provided for the groups to write down their ideas. The facilitators walk through all the groups, providing clarity to aspects that the group was not clear about. After 45 min, each group was allocated 15 min to present and elaborate on their topics. An additional 5 min was given for clarity seeking questions after each presentation. A voice recorder was used to record the presentations. Facilitators emphasised important points that groups should remember after each presentation.

### Data analysis

The eight steps of content analysis as indicated by Bengtsson (2016) were used to analyse the presentations. After the presentations, the facilitators collected all the flip charts and two transcribed the collected data while listening to the presentations from the video recorder. The two facilitators went ahead categorising similar data and assigning codes. This process was repeated until all collected data were coded. The facilitators submitted data to the third facilitator to re-check the narratives and coded data to ensure consistency. In the final step, the three facilitators reached a consensus on the coded data. From the transcribed data, the facilitators came up with three main themes and seven subthemes, as indicated in Table 1.

**TABLE 1:** Themes and subthemes that emerged during the presentation.

Themes	Subthemes
1. Use of Ubuntu philosophy to care for and prevent stigma and discrimination in people living with HIV/AIDS or TB	1.1. Being humane 1.2. Ensure privacy and confidentiality 1.3. Change of attitude and community behaviour
2. Practicing Ubuntu in preventing the spread of HIV/AIDS or TB	2.1. Awareness of HIV as a virus and TB as a bacteria 2.2. Strategies to prevent the spread of HIV/AIDS
3. Integrating Ubuntu in the management of HIV/AIDS or TB as a chronic condition	3.1. Volunteering to assist with love 3.2. Encourage disclosure of one’s status

HIV, human immunodeficiency virus; AIDS, acquired immunodeficiency syndrome; TB, tuberculosis.

### Measures to ensure trustworthiness

In this study, trustworthiness was adhered by credibility, dependability, transferability and confirmability (Enworo 2023). *Credibility* refers to confidence in the truth of the findings of the research study (Polit & Beck 2018). Credibility was achieved through prolonged engagement with the participants and their active participation in the 5-day workshop. Credibility, therefore, refers to the researcher making use of observations during the presentations made by the groups, the information contained in the flip charts, and voice recordings of the presentations to ensure and support triangulation. *Dependability* refers to evidence obtained if the study was repeated with the same questions with the same participants in a similar context; the findings would be the same (Houser 2018; Polit & Beck 2020). In this study, the facilitators indicate detailed information on how the workshop was conducted, including the participants and their presentations. *Transferability* is when findings can be transferred to other groups or settings. The researcher must provide enough information to allow judgements about the context of the data (Denzin & Lincoln 2021; Polit & Beck 2020). In this instance, if a similar workshop can be conducted using the same participants and the same materials used during the workshop, the facilitators might get the same information and results as indicated in this article. *Confirmability* refers to the steps taken by the researcher to demonstrate that the findings of two or more independent researchers achieve congruence. If other facilitators can repeat the same workshop with the same participants in the same context, they will get the same results.

### Ethical considerations

Approval to conduct the study was given by one public University of Pretoria, Faculty of Health Sciences Research Ethics Committee project number 465/2020. The community leaders of the village where the workshop was held gave their consent and the participants volunteered to be part of the workshop. They have given their verbal and written consent for this article to be written. Privacy and confidentiality regarding the presentations were maintained as the information presented was only known by the members who attended the workshop and with the authors. However, the groups were told that their presentations would be shared through publication as consent was sought for that. Anonymity was ensured by not mentioning the names of group members as the presentations were labelled by groups, e.g. group 1, 2, 3, etc. No harm was encountered by participating in the workshop; instead, participants benefited as they were empowered with knowledge.

## Results

Table 1 presents the results of the work presented by the community during the workshop. Of the eight topics that were presented, facilitators came up with three themes and seven subthemes.

### Theme 1: Use of Ubuntu philosophy to care for and prevent stigma and discrimination in people living with HIV/AIDS or TB

This was the first theme that emerged from the presentation; the theme was presented by two groups. From it, both groups emphasised four subthemes, which were to ensure humanity to people living with either HIV or those suffering from TB; ensuring privacy and confidentiality of their shared information, community change of attitude as they display negative attitude towards people living the two conditions and to offer them social support by initiating support groups and encouraging people living with the two conditions and their families to join the support group.

#### Subtheme 1.1: Being humane

The groups indicated that the community must show love, kindness and generosity when integrating and caring for PLWHIV/AIDS or TB. They must be made aware of what HIV/AIDS and TB are in order to reduce stigma and discrimination:

‘If the community is understanding HIV/AIDS & TB, it will be easy to lend a helping hand to those in need, for example, they will help each other by providing and preparing nutritious food when one does not have.’ (P1, G1, matriculant); (P2, G2, THCP)

One group in their presentation indicated that those affected by HIV or TB must be treated like other people to avoid discrimination and stigmatisation:

‘People living with HIV must be treated as another person without a virus, treat others the same way you will want other people to treat you.’ (P2, Group 2, THCP)

In support of what the group has reported earlier, another group in their presentation reported that trust is essential for those living with the conditions to be open. The group agreed by indicating that:

‘Those who care must instil trust and this will make them feel free to verbalise and talk freely about their problems.’ (P1, G1, matriculant)

#### Subtheme 1.2: Ensure privacy and confidentiality

In their presentation, the group indicated that the community must avoid discussing the patient’s status with other people without their consent, as it is a sign of gossip. For those who are aware of their status, such as CHCWs and traditional healthcare practitioners, to ensure the privacy and confidentiality of patients under their care:

‘In our group, we felt that if people in the community can stop talking behind the back of people living with HIV or TB and accept them as they are, it can reduce stigma and discrimination.’ (P1, G1, matriculant)

Both groups further indicated how confidentiality and privacy can be maintained by indicating that:

‘As community health care workers, we believe that we cannot share the information of our patients without their permission, as such, the community must receive this information including family members. By doing so, stigma and discrimination can be reduced.’ (P3, G1, CHCW); (P2, G2, THCP)

Group 2 continues and emphasised confidentiality and privacy as follows:

‘We believe that if a patient comes and consults and I am in the clinic, I have seen that the person came for treatment, I must not discuss his information with others, it is his information, and our community must be taught this.’ (P2, G2, THCP)

#### Subtheme 1.3: Change of attitude and community behaviour

In their presentation, the group reported that the community must be cautioned about its negative attitudes and behaviour that will indicate non-acceptance of people affected by HIV as this will be an indication of discrimination. When engaging with PLWHIV or TB, compassion and support must be practised at all times; this is another way of showing acceptance:

‘We feel that people in the community must stop badmouthing others, especially if one is aware of the status of people with the virus, embrace, associate with them, and involve them in whatever the community is doing.’ (P2, G2, THCP); (P5, G5, CHCW)

The community must be educated about Ubuntu values such as caring, love and respect, which must be displayed all the time when caring for PLWHIV or TB. Social support must be provided to families and PLWHIV as a sign of acceptance, hence preventing stigmatisation:

‘The community must avoid assuming that people who have lost weight or have signs of ill health are HIV positive, as this will lead to pointing fingers at affected people and thus resulting in stigmatisation.’ (P1, G1, matriculant); (P2, G2, THCP)

### Theme 2: Practicing Ubuntu in preventing the spread of HIV/AIDS or TB

From this theme, two subthemes emerged as presented in Table 1, where in their presentations, the groups indicated an issue of educating the community to be aware that HIV is only a virus that cannot be spread by touching people living with it. The groups further indicated how it can be prevented, for example, the use of condom. For TB, it must be known that the cause is bacteria in which its spread can only be prevented by adhering to treatment. The two subthemes are presented as follows.

#### Subtheme 2.1: Awareness of HIV as a virus and TB as a bacteria

From this theme, the group indicated that the community must first be aware of HIV as a virus and TB as a bacterium that can spread from one person to another. They are to be encouraged to go for pretest counselling so that they know their HIV status and for those with signs of TB to go for screening so that early diagnosis can be carried out, hence this will prevent the spread to other people:

‘As a group, we feel that the community are to be taught first of HIV as only a virus so that they treat other people well. People in the community should be given more information on HIV so that they can be tested and know themselves.’ (P3, G3, CHCW)

‘When we go around the villages, we must teach the community about HIV so that they go for testing and also about TB so that people know the signs and symptoms so that they go for screening and testing to prevent the virus or bacteria from spreading.’ (P4, G4, CHCW); (P5, G5, matriculant)

The group elaborated in their presentation by indicating that respect must be shown when engaging and giving advice to PLWHIV/AIDS or TB so that they do not transmit the virus to others purposefully:

‘When teaching them, we must show them respect and verbally explain that even living with the condition, they are still loved by the community and their families. Therefore, we must advise them to prevent the spread of the virus with love.’ (P3, G3, CHCW)

#### Subtheme 2.2: Strategies to prevent the spread of HIV/AIDS

From the presentation, one group indicated that those who are sexually active should avoid having multiple partners, sleeping around and engaging in unprotected sex, as they will be putting their lives and their loved ones at risk:

‘People who are involved in sexual activities should avoid sleeping with a lot of people. People to use condoms as it will help prevent the spread of HIV.’ (P4, G4, CHCW)

The other two groups agreed by indicating other strategies such as abstinence, correct use of condoms, and avoiding the exchange of needles and razor blades. These strategies were emphasised during the presentation as one of the pieces of information to be distributed to the community:

‘The community must be taught how to wear a condom. This has to be done with respect, as other people are older than us. As some of us are traditional healers, we will teach others not to share razors, and even older people like to save money by using one razor to remove the hair of all people in their homes.’ (P4, G4, matriculant)

‘Those who use drugs, avoid passing the needle from one person to another as it will increase the spread of the virus. And once again, the community must be made aware of this information that we have learnt, it will reduce the passing of HIV. And for TB, they must cough through their elbow as this will prevent droplets from spreading to others.’ (P7, G7, CHCW); (P6, G6, THCP)

The issue of sharing what they have learnt with others was also empathised, as it will assist in promoting case identification, treatment adherence and follow-ups to prevent the spread of HIV and TB:

‘We have learnt a lot in the past three days, with this information we will teach others in the community so that people can identify other members who are sick and go to the clinic to get treatment and prevent the spread of tuberculosis.’ (P4, G4, CHCW)

### Theme 3: Integrating Ubuntu in the management of HIV/AIDS or TB as a chronic condition

From this theme, the groups indicated that when caring for PLWHIV/AIDS or TB, it is necessary to treat them equally as other people. Volunteer and treat them with love and acceptance. When managing their conditions, to encourage them to disclose their status to their family members, as this will relieve their stress of thinking about their condition:

#### Subtheme 3.1: Volunteering to assist with love

One of the groups indicated that they would volunteer to help with activities such as house chores, especially those who are sick and unable to assist themselves. PLWHIV/AIDS or TB must be assisted with love and by offering continuous counselling from whatever problems they might be experiencing and their family. In this way, stress will be reduced:

‘To practice ubuntu, patients who are sick must be shown love for example they are to be assisted, they cannot go hungry while we can cook for them, they cannot leave in a dirty area when we can clean their homes so that they are comfortable.’ (P8, G8, matriculant)

In their presentation, another group indicated the issue of reminding their patients to take their treatment on time and to eat nutritious balanced meals before treatment:

‘We must teach their family members to set a clock to remind them to take their treatment on time and eat food to build their body and health. This is important if we want them to improve. There must always be someone to remind them to take treatment.’ (P4, G4, CHCW); (P8, G8, THCP)

#### Subtheme 3.2: Encourage disclosure of one’s status

The groups indicated the issue of encouraging them to disclose their status to their family members so that they can support them; this included the issues of joining support groups so that they talk freely about their condition, learn from others and support each other:

‘They are support group in various clinics, so we must make them aware of these groups so that they can join. In the support group, people are free to talk about their conditions. But for them to do so, they must be ready to come out for people to know their conditions.’ (P7, G7, CHCW)

‘We must encourage them to tell their families about their conditions. This will help them to be free. And joining a support group can make them feel strong and supported.’ (P8, G8, matriculant)

## Discussions

### Use of Ubuntu philosophy in caring and preventing stigma and discrimination among people living with human immunodeficiency virus and those suffering from tuberculosis

From the presentations, it was evident that humanity must be practised when caring for people with HIV/AIDS or TB. Humanity refers to the qualities of displaying generosity or kindness towards another human being, as such the group who presented this theme indicated that the community must first understand the concepts ‘HIV/AIDS or TB’ and what it entails. The group continued by indicating that PLWHIV must be treated like another person without a virus in that way stigma and discrimination might be prevented. Some authors indicated the concept of humanity and integrating it into Ubuntu as ‘treat others the same you will want other people to treat you’ (Resnik 2018, 2022). ‘I am a person because you are, I am because I share and participate’ and ‘I am because of others’. As such, Ubuntu as an African philosophy expresses humanness in the values of compassion, solidarity, harmony, consensus, hospitality, sympathy and sharing, among others (Chigangaidze, Matanga & Katsuro 2022; Mupedziswa, Rankopo & Mwansa 2019). The groups indicated that the community must avoid discussing the patient’s status with other people without their consent as it is a sign of gossip; this will ensure confidentiality and privacy. The two ethical principles are practised in healthcare sciences to promote the dignity of patients, as well as to keep their conditions privately, especially from people who do not care for such patients (George & Bhila 2019; Parsons 2021). The community must be educated about Ubuntu values such as caring, love and respect, which must be displayed all the time when caring for PLWHIV or TB. The same results were indicated in a study conducted with university students where they indicated that they must show love and respect when caring for their patients in the hospital or in the community (Rasweswe et al. 2024). In their presentation, change of attitude and social support was encouraged and must be provided to families and PLWHIV or TB as a sign of acceptance, thus preventing stigmatisation. The community should be warned regarding their negative attitudes and behaviour towards PLWHIV, as this will be an indication of discrimination. If stigmatisation and discrimination are not addressed, it can lead to depression and anxiety in PLWHIV or TB (Dejman et al. 2017; Fauk et al. 2021).

### Practicing Ubuntu in preventing the spread of HIV/AIDS or TB

From their presentation, the groups indicated that the community must first be made aware of HIV as a virus and TB as a bacterium that can spread from one person to another. To prevent the spread of HIV and TB by those already infected, the emphasis on compliance to treatment in order to reduce the spread of the two conditions was emphasised (Sheehan et al. 2020). The groups further indicated that they would encourage the community to go for pretest counselling so that the community knows their HIV status and for those with signs of TB to go for screening so that early diagnosis can be made, hence this will prevent the spread to other people. In a study conducted by Hsu and Rakhmanina (2022), the authors indicated that pretest counselling and testing must be performed from youth at 15 years and older. After an initial test, it must be performed annually for those at risk, sexually active at 3–6 months for males who have sex with another male (Hsu et al. 2022; Stupiansky et al. 2017). Strategies such as abstinence, proper use of condoms and avoiding needle sharing were emphasised during the presentation as one of the pieces of information to be disseminated to the community. Preventive measures were also mentioned in a study conducted by Santos et al. (2021) and Hsu et al. (2022).

### Integrating Ubuntu in the management of human immunodeficiency virus/acquired immunodeficiency syndrome as a chronic condition

From the presentation, the groups indicated that when caring for PLWHIV/AIDS or TB, it is necessary to treat them equally as other people. Show love and acceptance when managing your condition, as this will relieve the burden of thinking about your condition. Similarly to what was presented by Group 2, the presenters indicated the issue of reminding their patients to take their treatment on time and eat nutritious food before taking treatment. The group indicated the challenge of encouraging them to disclose their status to their family members so that they can support them; this included the issues of joining support groups so that they talk freely about their condition, learn from others and support each other even mental (Santos et al. 2021; Thompson et al. 2021). According to Mbokazi et al. (2023) incorporating Ubuntu and linked African support theories into current treatment burden models will allow better understandings of patient collective support and can inform the development of context-specific social health interventions that fit the needs of people living with chronic conditions in African settings. In support of the presentation carried out by this group, the philosophical value of Ubuntu (sharing, generosity, care and help) was indicated (Chigangaidze et al. 2022).

## Conclusion

Based on the presentations made by the eight groups, it was evident that the participants were empowered with the knowledge of integrating Ubuntu that they will share and implement when caring, engaging and managing PLWHIV/AIDS or TB. As such, stigmatisation and discrimination will be reduced. The community will accept PLWHIV and those suffering from TB, help them when there is a need, and treat them with dignity and respect. The philosophical values of Ubuntu were instilled in the participants and will be displayed when working with people affected with HIV/AIDS and TB and their families. Privacy and confidentiality will be practised, and PLWHIV or TB will be encouraged to disclose their status to their family and join support groups. As such, it is vital that members of the community be aware about Ubuntu so that they apply it in their daily interaction irrespective of their conditions.
